# Use of Humidity Controlled Quartz Crystal Microbalance with Simultaneous Grazing Incidence Small Angle X-ray Scattering to Investigate the Self-assembly and Energetics of Lipid Thin Films

**DOI:** 10.1021/acs.langmuir.4c05158

**Published:** 2025-04-16

**Authors:** Jack Macklin, Christian Pfrang, Paul Wady, Wanli Liu, Ruaridh Davidson, Adam Milsom, Adam Squires

**Affiliations:** 1 Department of Chemistry, University of Bath, South Building, Soldier Down Ln, Claverton Down, Bath BA2 7AY, U.K.; 2 School of Geography, Earth and Environmental Sciences, University of Birmingham, Edgbaston, Birmingham B15 2TT, U.K.; 3 Diamond Light Source, Diamond House, Harwell Science and Innovation Campus, Didcot OX11 0QX, U.K.; 4 School of Chemistry, University of Bristol, Cantock’s Close, Bristol BS8 1TS, U.K.

## Abstract

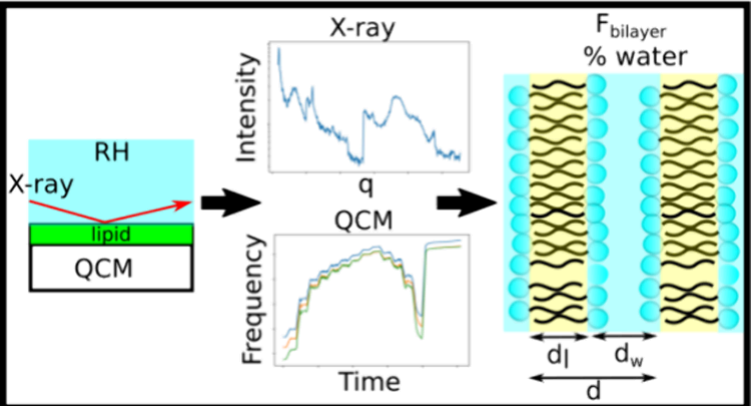

We present a novel method of analyzing lyotropic liquid crystal mesophases—self-organized amphiphile-water nanomaterials—using *in situ* grazing-incidence small-angle X-ray scattering (GI-SAXS) on a quartz crystal microbalance (QCM) in a controlled humidity environment. This combination simultaneously gives nanostructural dimensions and phase symmetry (through SAXS), compositional data (% water by weight, from QCM data), and water activity within the sample (from the equilibrium relative humidity above the film), as the sample film takes up and releases water during humidity sweeps. Analysis of the combined data provides immediate access to information typically built up from experiments on multiple individual samples prepared at different fixed compositions. Our approach greatly reduces the required sample quantities and preparation time while avoiding issues with sample-to-sample variations thanks to the collection of the multiple parameters simultaneously. It also extends the accessible range to the low water content region of the phase diagram, which is harder to access by fixed composition measurements and is highly relevant to coatings and powders exposed to ambient humidities. Here, we present data on dimyristoylphosphatidylcholine/water lamellar phases. Our calculated bilayer thickness and interbilayer repulsion values show good agreement with published data obtained from multiple individual samples, and we clearly demonstrate the ability to extend to lower water contents.

## Introduction

Amphiphilic molecules such as biological lipids and industrial surfactants self-organize when mixed with water into materials known as lyotropic liquid crystalline mesophases. These mesophases possess a range of different nanostructures, ranging from bilayers and micelles to complex 2D and 3D arrays. The arrangement greatly impacts physicochemical properties such as diffusion, viscosity and optical transparency, and therefore biological, pharmaceutical, industrial or environmental behavior.^1,2^

To understand and characterize this self-organization, scientists typically study “fixed hydration” samples, where known masses of amphiphile and water are carefully mixed. The samples are analyzed by X-ray or neutron scattering to give information on the symmetry and unit cell dimensions of the periodic nanostructures in the mesophase, for example, the spacing of a stack of bilayers in a lamellar phase. The complete data set showing the nanostructure as a function of water content typically takes 0.1–1 g of material. The data set can be used to build up phase diagrams, or to determine geometric information on molecular packing.^3,4^

A second class of experiment gives insights into the energetics and forces of interactions between nanomaterials; for example: interbilayer repulsion, or monolayer bending energy.^5,6^ This is achieved by allowing the amphiphile-water mixture to gain or lose water in equilibrium with water of controlled chemical potential: from applied hydrostatic or osmotic pressure, or from a vapor of known relative humidity (RH).

In addition, scientists have used SAXS to study films or levitated droplets in equilibrium with known relative humidity as a focus in itself, to understand the humidity response of amphiphile self-organization in a particular context, for example, to understand atmospheric phenomena.^7^ In each case, the data alone do not track the changing water content directly, and for further calculations (e.g., on energy) it is often inferred indirectly by correlating the unit cell dimensions with a fixed hydration (“gravimetric”) data set.^7,8^

Quartz crystal microbalances (QCM) have recently emerged as a sensitive tool for monitoring changes in mass as materials adsorb onto a substrate. The substrate is typically gold, coated onto a vibrating piezoelectric quartz crystal, and an increase in adsorbed mass causes a proportional decrease in resonance frequency, through the Sauerbrey equation.^9^ This equation strictly only applies for a “rigid” coating. For viscoelastic materials such as proteins or other biomolecules, the fundamental QCM frequency alone no longer gives quantitative mass data, although the qualitative changes have allowed widespread use to monitor the adsorption process and its time scale, in biosensors and other applications.^10−13^ However, scientists have recently shown that, with sufficiently thin films, data from multiple vibrational overtones can be extrapolated to allow quantitative mass measurement from viscoelastic materials.^14−18^

Here, we demonstrate for the first time mass measurements on lipid films using multiple overtone QCM measurements, while simultaneously collecting X-ray scattering patterns. By varying the relative humidity (RH) that the samples were exposed to, we could therefore determine what the water content of the film was at any given point and relate it to the features of the film that are obtained from the GI-SAXS patterns, to give insights previously requiring multiple fixed-hydration mixtures.

## Experimental Section

### Materials

Dimyristoylphosphatidylcholine >99% purity (DMPC) was purchased from Avanti Polar Lipids (USA) and used as provided.

Samples were prepared by making stock solutions of DMPC in ethanol at 5 wt % via dissolution prior to spin coating.

### X-ray Parameters

We used the Xeuss 3.0 (Xenocs, France) “LabSAXS” instrument at the Diamond Light Source to perform our measurements. The X-ray source was a Gallium MetalJet (Excillium) which has a K_α_ emission of 1.3 Å (9.2 keV). The sample to detector distance was 285 mm, calibrated with silver behenate. The beam size was 250 × 250 μm, and all patterns were collected with an exposure time of 55 s.

### QCM

An openQCM Q^–1^ with Dissipation Module (openQCM, Italy), was used as our measurement device. This was modified with custom firmware developed by the openQCM team to allow it to be used with the openQCM Next (openQCM, Italy) software, enabling the measurement of multiple overtones simultaneously.

The 14 mm quartz holder USB module (openQCM, Italy) was used as the sample mounting stage to allow for the sensor wafer to be exposed and accessible with X-rays.

The sample environment for simultaneous GI-SAXS/QCM is presented at the start of the Results and Discussion. The humidity control was as in Milsom et al.^19^ In brief, this technique involves two pumps operating in tandem. One of these pumps pushes dry air, while the second first directs the air through a bubbler to humidify it before delivery. By controlling the rates of these two pumps, a specific RH value can be targeted. Milsom et al. used air directly from the laboratory as their dry air source and were therefore limited to a minimum humidity equal to that of the laboratory. However, we first pumped dry air from a compressed air source into a box in which the dry air pump was placed, before pumping that air into our RH controlled system at a known rate, allowing us to access humidities below that in the laboratory.

### Thin Film Preparation

The thin films were prepared by spin coating onto 5 MHz quartz QCM wafers with gold electrodes (openQCM, Italy) for 45 s at 2000 rpm. Prior to spin coating, the QCM wafer was manually cleaned with a lint-free lens tissue soaked in ethanol, and then rinsed with a spin coat step using 1 mL ethanol dripped slowly over 60 s at 2000 rpm. The spin coating solution was 5 wt % DMPC in ethanol. Following spin coating, all ethanol has evaporated from the surface, leaving a layer of pure lipid.

## Results and Discussion

**Figure 1:**
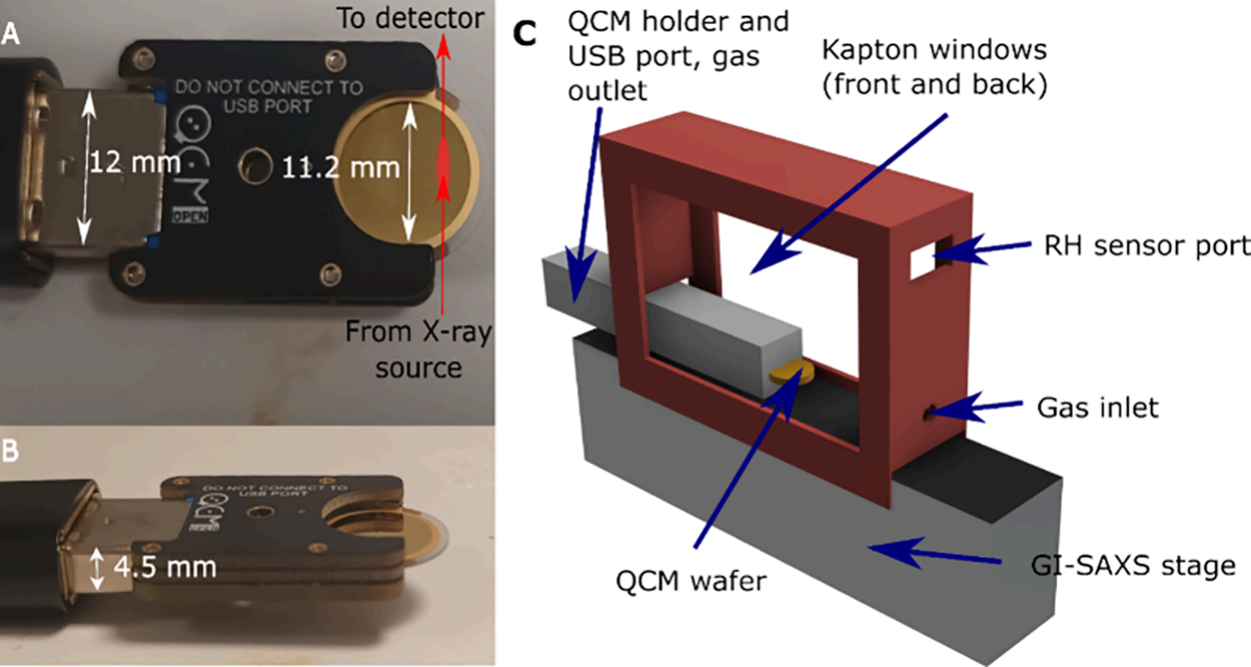
Mounting of the QCM chip in the USB module and its connection via USB extension cable, as well as the humidity chamber designed for use with the Xenocs diffractometer. Also shown are some key dimensions of the system and the path and footprint of the X-rays on the QCM chip (red arrows and red oblong respectively, beam traveling in the plane of the page). The sample is spin-coated across the whole of the QCM chip, but the QCM signal comes only from the section covered by the gold electrode. (A) Top-down view. (B) Angled side view. (C) Humidity chamber.

The experiments were carried out with the sample coated on a QCM chip, held in a controlled humidity chamber (Figure 1C). To ensure that the air gap between the X-ray source and the detector vacuums was as small as possible, we had to minimize the width of our sample chamber. To do so, the main openQCM Q^–1^ module was kept out of the chamber and the USB module was connected via a 50 cm USB extension cable. The USB module does not fully encircle the QCM chip, and so allows the incident beam to impinge on the sample with a footprint as shown in Figure 1A. The chamber was a 3D printed box (31 mm × 99 mm × 60 mm, V = 190 mL, Figure 1C) with notches to fit over the Xeuss diffractometer (Xenocs, France) sample stage snugly. There were large windows cut out of it that were sealed with Kapton foil, an X-ray transparent polymer. There were additional holes for the relative humidity-controlled gas flow and for the USB cable to pass through. The chamber was designed to fit perfectly flush with the GISAXS stage installed in the Xeuss 3.0 (Xenocs, France) instrument at the Diamond Light Source (“LabSAXS” instrument). Adding the USB cable to reduce the cell size improved the quality of the collected X-ray patterns. Although the slight increase in electrical noise introduced into the QCM data meant we could not resolve the ninth and often the seventh overtones, we demonstrate here that we could obtain meaningful mass data from the first, third and fifth overtones.

**Figure 2:**
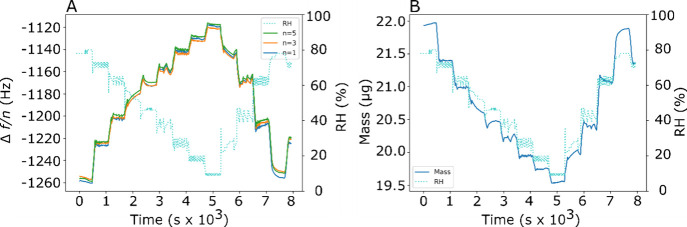
(A) Normalized frequency shift and the RH vs time for a sample of DMPC spin coated onto a QCM sensor. The fundamental frequency and the third and fifth overtones are shown. The RH is shown with a dotted light blue line, the fundamental frequency with a solid dark blue line, the third overtone with a solid orange line and the fifth overtone with a solid green line. (B) The variation of the mass of a DMPC film over time as the RH is varied. The mass is shown by a solid blue line and the RH by a dotted turquoise line. The approximate dry lipid mass is 19.5 ± 0.2 μg.

We present data on the lipid DMPC (dimyristoylphosphatidylcholine), previously shown to form a lamellar phase in water or a humidified environment.^20−22^ We performed humidity ramps between 5% and 80% RH obtaining simultaneous QCM and GI-SAXS data. Figure 2A shows the change in frequency response of several overtones of the QCM sensor over time, and the corresponding RH that the sample was exposed to. The frequency is plotted as the normalized frequency shift, i.e. the difference between the measured frequency and the frequency of the bare wafer divided by the order of the overtone, to allow for the different overtones to be compared. We see that variations in the RH cause a reversible change in the recorded frequency. We also see that the three measured harmonics are close to one another. This is characteristic of a sample close to the Sauerbrey regime; the slight decrease in frequency with higher overtone suggests that the sample is slightly viscoelastic.^16^ In order to estimate the mass, we use the model for a single viscoelastic film in air:^16,18^
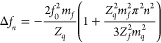
1where Δ*f*_*n*_ is the overtone normalized frequency shift (Δ*f*_*n*_ = Δ*f*/*n*), *f*_0_ is the fundamental resonance frequency of the bare chip, *Z*_*q*_ is the acoustic impendance of quartz, *n* is the number of the overtone, *m*_*q*_ is the areal mass density of quartz and *m*_*f*_ is the areal mass density of the film. The mass can then be estimated by extrapolating to n = 0. We chose to use this model because, in the simplest case where there are no differences between the normalized frequency shifts of the various overtones, this model simplifies to the Sauerbrey model, and should differences arise in more complex viscoelastic cases, this gives a better accuracy for the mass calculation.^15,16,18^

Figure 2B shows the variation of the mass over time of a thin film of DMPC as the relative humidity is varied. The mass change is reversible, showing that the process is thermodynamic, with the lipid film in equilibrium with the surrounding water vapor. The lowest mass, which we assume approximately corresponds to the dry mass of the lipid alone (all water having evaporated), is 19.5 ± 0.2 μg. We have assumed that this corresponds to the mass of the lipid alone as the mass measurements plateau around this value. Over the diameter of the QCM electrode this mass corresponds to a film thickness of 198 ± 2 nm, assuming a density of 1 g cm^–3^ for the lamellar phase, as in Parsegian et al.^23−25^ This film thickness is of the order of magnitude that we would expect from thicknesses of similar lipid films spin-coated from ethanolic solutions.^26^ The mass increases up to 22.0 ± 0.2 μg at high RH, corresponding to an uptake of 2.5 ± 0.2 μg water, from *m*_*water*_ = *m*_*sample*_ - *m*_*lipid*_, assuming that *m*_*lipid*_ = 19.5 ± 0.2 μg.

**Figure 3:**
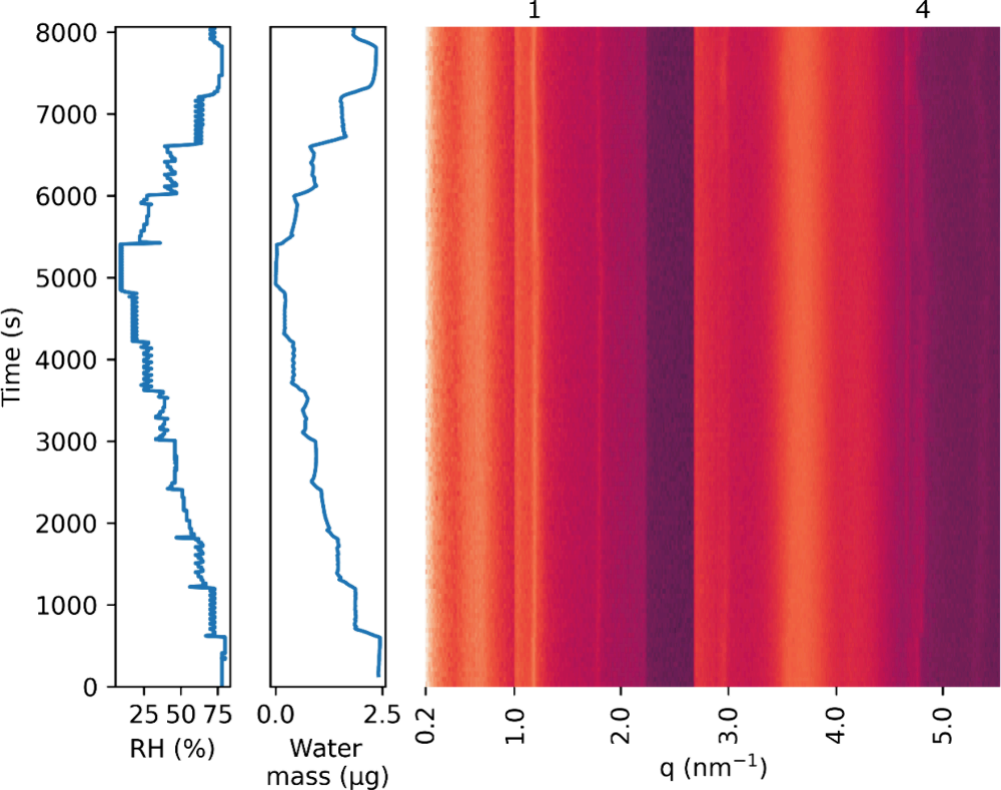
Variation over time of relative humidity, water mass in the film and X-ray scattering pattern for a sample of DMPC spin-coated onto a quartz QCM sensor. The heatmap on the right shows the scattering patterns collected over time, with bright colors corresponding to high intensity and dark colors to low intensity. All patterns were collected with an exposure time of 55 s. The 1st and 4th order lamellar peaks are labeled. There are artifacts due to reflected peaks (around 1.8, 2.95, and 5.3 nm^–1^), Kapton windows (3.8 nm^–1^), and the detector gap (2.4 nm^–1^). The approximate dry lipid mass is 19.5 μg.

Our combined GI-SAXS/QCM experiment allows us to probe changes in nanostructure accompanying this water uptake and loss. Figure 3 shows the relative humidity and water mass data combined with the simultaneous reduced GI-SAXS data for the humidity sweep experiment. The reduced 1D SAXS data sets are combined into a heatmap plot as in Milsom et al.,^7,19^ where each horizontal row corresponds to the scattering pattern corresponding to that relative humidity, with intensity shown as color variation. The GI-SAXS plot shows the first and fourth order reflections from the lamellar phase adopted by the DMPC, at *q* values of approximately 1.2 and 4.8 nm^–1^ (labeled in Figure 3). This is consistent with what has been previously observed for DMPC, both as a bulk solution and as thin films.^20,23,27^ The second and third order reflections cannot be seen, as they coincide with the detector gap and the reflection from the Kapton windows respectively at approximately 2.4 and 3.6 nm^–1^ (see pattern from empty chamber, SI, Figure S2). Further signals at *q* ≈ 1.8 and 5.3 nm^–1^ arise from diffraction from the specular reflected beam; a series of patterns showed these peaks to move position on varying the incident angle (SI, Figure S1), while the first and fourth order peaks at 1.2 and 4.8 nm^–1^ did not. We therefore concluded that the latter two were diffraction from the incident beam and used them to characterize the lamellar dimensions in further analysis.

We observe a dependence of the *q* values at which the sample scatters on the relative humidity to which the sample is exposed. Specifically, the *q* value increases as the sample dries out, corresponding to a reduction in the repeat distance of the unit cell, and *vice versa*. This change in the scattering vector is particularly visible for the fourth order scattering at *q* ≈ 4.8 nm^–1^ in Figure 3. From this, we can see that the water taken up by the system is interacting with the bilayer stack, causing the stack to swell by increasing the distance between consecutive lipid layers. We can quantify these changes from our combined data set. The repeat spacing *d* of a lamellar phase corresponds to the combined thickness of one lipid bilayer *d*_*l*_ and a layer of water, *d*_*w*_:^25^
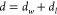
2

The value of *d* can be calculated from the position of the first reflection *q*_100_ using^28^
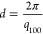
3

Determining the separate thickness contributions to *d* from the water layer and lipid bilayer requires the corresponding water/lipid ratio within the sample, which we obtain here using simultaneous QCM data. The water content corresponding to each relative humidity is given by
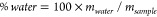
4

**Figure 4:**
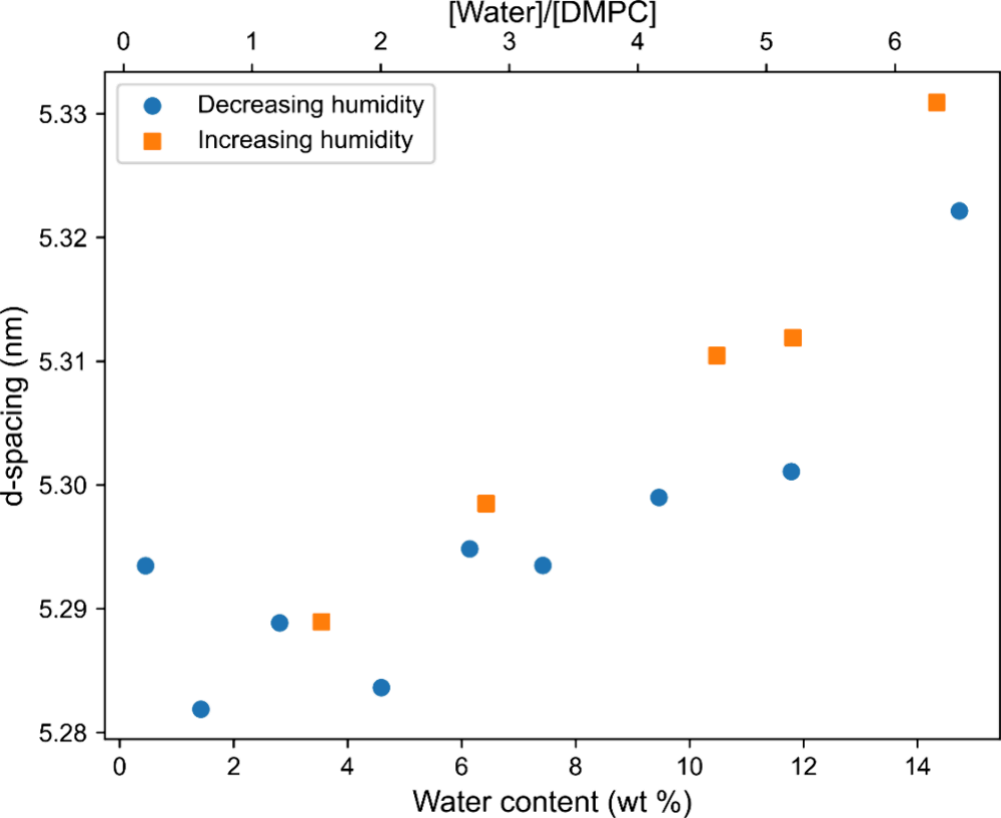
Variation of the *d*-spacing of the lamellar phase of DMPC as the water content is varied, as measured by simultaneous GI-SAXS and QCM. The data were collected both as the RH was decreased (blue circles) and as the RH was subsequently increase (orange squares). The errors are much smaller than the data points and are therefore not visible.

Figure 4 shows a plot of the *d* spacing (from GI-SAXS) against water content (from QCM). In previous literature, this data set was built up using a series of fixed-hydration samples.^28−30^ From here, we calculate the thicknesses of the water layer and of the lipid bilayer as follows:^25^
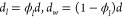
5
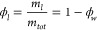
6where ϕ_*l*_ and ϕ_*w*_ are the lipid and the water mass fractions, respectively.

The calculation assumes the density of our liquid crystal to be 1 g cm^–3^ as in a previous work by Parsegian.^25^Figure 4 shows the values calculated for the *d*-spacing obtained as the humidity was decreased and then subsequently increased on the same sample. We see little difference between the two directions, showing that there were no history effects. Furthermore, the initial and the final values obtained from the humidity extrema agree (both in the case of the mass and the *d*-spacing). This agreement further confirms that the sample is under thermodynamic control and does not exhibit history effects. Additionally, this rules out beam damage effects from the exposure to the X-rays over the course of the measurements.

**Figure 5:**
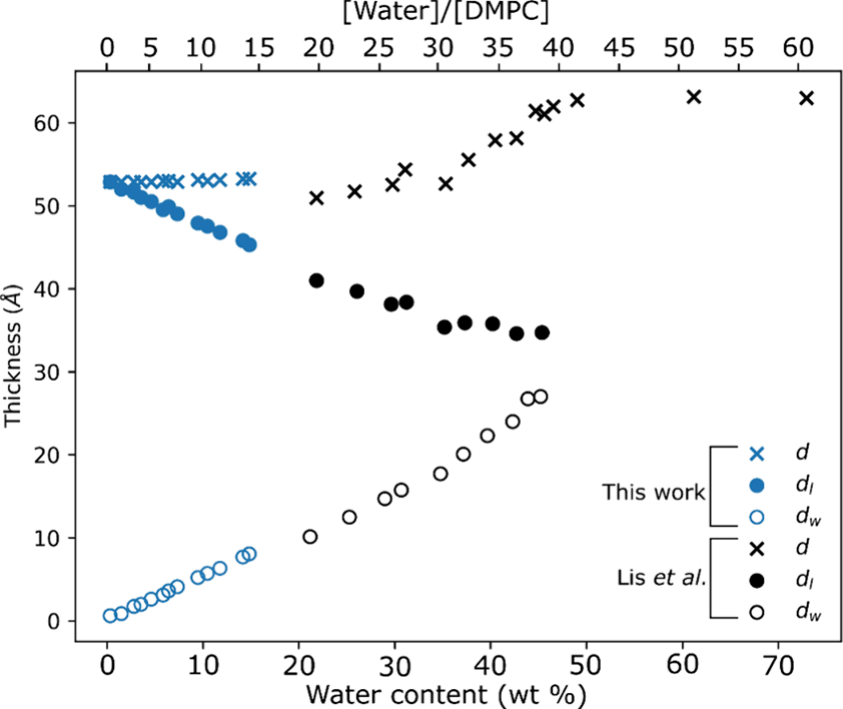
Water (open circles) and DMPC lipid layer (filled circles) thicknesses, along with the total lamellar stack thickness (crosses) calculated in this work (blue, smaller, low water content) compared to previous work (black, high water content) carried out by Lis et al.^6^ as a function of water content. Our work was carried out at 25 °C and Lis et al. carried out their measurements at 27 °C.^6^ Adapted with permission from ref (6). Copyright 2025 Elsevier.

Figure 5 shows how the data we obtained in these experiments compare with prior work by Lis et al.^6^ on DMPC. The data collected with our new technique of combining GI-SAXS with simultaneous QCM measurements aligns well with previous data collected using a series of fixed hydration samples, extending the observed trends to lower water contents. The slight discrepancy could be due to a difference in temperatures at which the measurements were performed; while our sample was held at 25 °C, Lis et al. worked at 27 °C.^6^

Our approach brings two key advantages. First, it requires much less sample: a series of separate fixed hydration samples would require approximately 0.1 g – 1 g total,^22^ while our experiments were performed on a single spin-coated film using approximately 2 mg lipid. Second, it takes much less time: fixed hydration gravimetric samples typically require an overnight equilibration step,^22^ while our experiments were conducted in a single 2 h sweep – the thin film allowed rapid equilibration; the QCM data in Figure 2 provide validation, and demonstration that in fact the equilibration is very fast, and likely limited by the time of changing humidity by our instrumentation.

**Figure 6:**
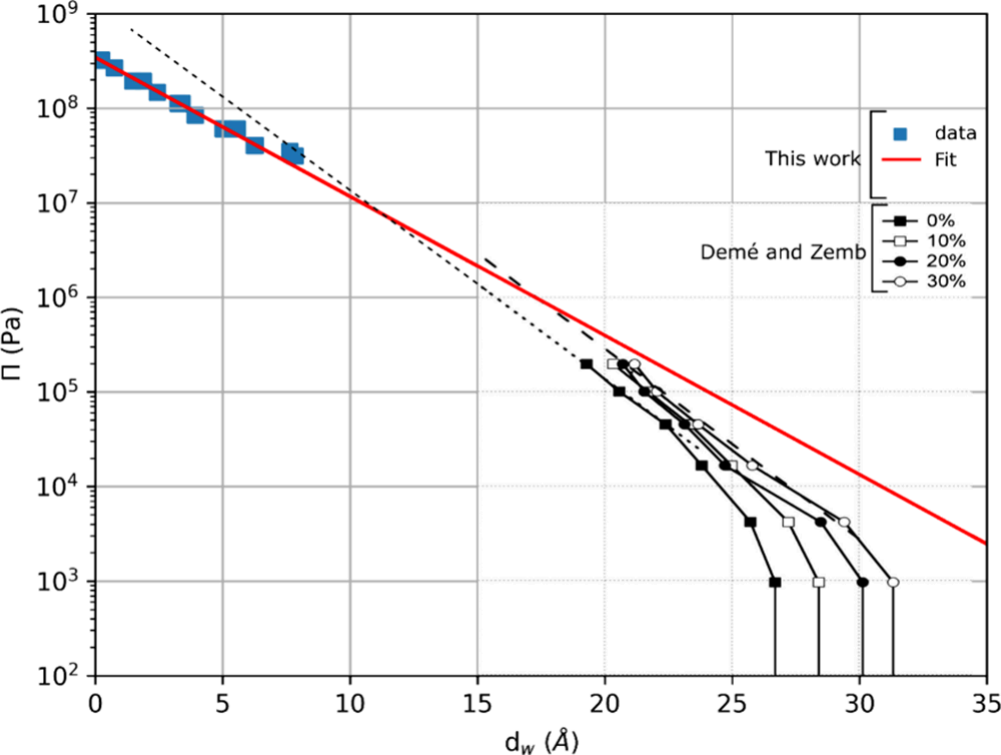
Osmotic pressure vs the water layer thickness for DMPC. Our data (blue, closed squares) and its fit (solid red line) is presented against data gathered by Demé and Zemb, who carried out the measurements for different concentrations of sugar in the system (the different black and white symbols at higher water layer thickness and black lines). The fits are from an exponential function of form Π = *P*_0_ + *Ae*^–*d*_*w*_/λ_*w*_^.^31^ Adapted with permission from ref (31). Copyright 2025 Elsevier.

The use of chemical potential information from the equilibrium relative humidity gave us the opportunity to study the forces at play between DMPC bilayers. To do this, we calculated the osmotic pressure Π that is exerted on the system by a given RH:^23^
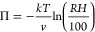
7where *k* is Boltzmann’s constant, *T* is the temperature, and *v* is the volume of a water molecule. Figure 6 shows how the osmotic pressure, Π, varies with the water layer thickness, showing the effect of pressure on the bilayer swelling. We compare the data that we have gathered to the findings of Demé and Zemb who studied the effect of osmotic pressure on the water layer thickness of DMPC in the presence of sugars.^31^ We see that our data fit well with previous measurements, although there is a slight difference in the gradient of the exponential function fits to the data. The exponential parameters calculated are ln(*P*_0_) = 19.50 ± 0.05 and λ_*w*_ = 2.67 ± 0.17 *Å*, where λ_*w*_ is the Debye–Hückel decay length, the length over which electrostatic interactions are expected to play a role. These parameters have previously been measured to be in range of 10 < ln(*P*_0_) < 19 and 1.96 *Å* < λ_*w*_ < 2.6 *Å* depending on the measurement method.^23,31^ As with the measurements of the water thickness and osmotic pressure, our data fit very well with previously published data obtained with multiple samples, validating our method.

Recently, Schlaich et al. published a predictive theory of the decay length of the hydration force for dipalmitoylphosphatidylcholine (DPPC), a lipid closely related to DMPC.^32^ In this work, they found λ_*w*_ for DPPC to be between 0.22 and 0.21 nm depending on whether the fluid lamellar or the lamellar gel phase was formed. The slight discrepancy between these values and those that we calculated in this work is likely due to differences between DMPC and DPPC. On the other hand, the similarity between both sets of results suggests that the predictive model would be viable if it were applied to DMPC specifically. One feature that is predicted by Schlaich et al.’s model is a plateauing of the osmotic pressure exerted by the system as the water layer thickness, *d*_*w*_*,* decreases even further, below ∼ 1 *Å*.^32^ We did not obtain measurements for multiple points with *d*_*w*_ < 1 *Å*, however, and we therefore cannot comment on the accuracy of the prediction of this plateau by the model.

The use of relative humidity has previously been published as a method to control water chemical potential, and therefore obtain force data between bilayers.^6^ Other approaches are hydrostatic pressure with a semipermeable membrane, and osmotic pressure achieved using polymer solutions, as in the work by Demé and Zemb (Figure 6).^31^ Relative humidity brings a number of advantages: it requires much less sample and less complex equipment than direct hydrostatic pressure; and it gives a direct measure of water chemical potential, avoiding potential errors introduced by use of polymers.^6^ However, our method is limited to very high osmotic pressures. Indeed, as one approaches 100% RH, very small changes in temperature can have extremely large effects on the osmotic pressures, with a 0.01 °C difference causing a sufficient change in osmotic pressure to cause around a third of the water to be removed from a bilayer if the RH is above 99.9%, making it very difficult to use this technique at humidities approaching saturation.^23^ It can therefore be seen as complementary to previous methods, and more suitable for probing lower water content samples.

In our experiments, simultaneous GI-SAXS and QCM accompanying the chemical potential information obtained from relative humidity provides further advantages over approaches using polymer-induced osmotic stress: in addition to reduction in sample quantity and equilibration time, as discussed above, the fact that the different data are obtained on the same sample avoids errors introduced by requirements to separately obtain a fixed-hydration gravimetric data set, to correlate osmotic stress scattering data with water content.^6^

## Conclusions

We have demonstrated, for the first time, simultaneous determination of water content, nanostructure, and water activity, using a QCM-GI-SAXS controlled humidity device. This approach simultaneously gives two data sets, each of which would previously have required preparation of a series of samples. These data sets are first, nanostructure vs water content, previously obtained from experiments on fixed hydration gravimetrically prepared mixtures; and second, data correlating both nanostructure and water content with water activity, which would previously have required cross-correlation between two series of samples. Our approach therefore gives two key advantages: first, in practical terms, a saving in sample requirement by two orders of magnitude, as well as time saving in sample preparation and equilibration; and second, in data quality, by reducing errors introduced by correlation between data sets and preparation of gravimetric samples.

For the lamellar phase we present here, from the nanostructure vs water content data, we determined bilayer and water layer thickness; and from the complementary water chemical potential data we determined interbilayer repulsion forces. Our results show consistency with published data.

These two types of analysis have been used more widely in the study of a wide range of nonlamellar lyotropic phases, including inverse hexagonal and bicontinuous cubic phases. Fixed hydration scattering data sets have been used to build up phase diagrams, and to determine lipid geometry in the curved monolayers of these phases,^33−35^ while controlled chemical potential osmotic stress experiments have been used to measure monolayer bending elasticity.^5,33^ Our approach therefore has the potential to bring the advantages mentioned above to answer this wider range of questions.

The particular features of our technique suggest two areas for research that the current study will enable. The first is the study of lipid or surfactant samples where only very small quantities are available. Our approach will, for the first time, allow insights into the structures and energetics of lyotropic nanostructures on such materials, prohibited by the large sample quantities required by previous experimental methods using fixed hydration samples. The second is in low water content lyotropic systems, which suffer more from difficulties in sample homogenization and equilibration in previous approaches with gravimetrically mixed samples. Low water content lyotropic systems are more common in films or aerosol particles that can lose water to equilibrate with surrounding ambient humidity. However, due to our assumption of the dry mass being the same as the mass we recorded at 5% RH, we may be somewhat underestimating the water content as the last few waters around a headgroup are likely to be difficult to remove. Future experiments using thermogravimetric analysis may rule this out.

Surfactant molecules are found in a wide range of aerosol types and contexts, including marine, anthropogenic, exhaled and pharmaceutical aerosols, where their behavior is the focus of considerable recent research,^36−42^ and their hygroscopicity is key to understanding their physical behavior and environmental or pharmaceutical impact.^1,7^ Our approach is therefore likely to enable research in a wide range of scientific fields.

## Supporting Information Available

Additional experimental details used for the assignment of peaks (PDF)

## References

[ref1] Pfrang C.; Rastogi K.; Cabrera-Martinez E. R.; Seddon A. M.; Dicko C.; Labrador A.; Plivelic T. S.; Cowieson N.; Squires A. M. Complex Three-Dimensional Self-Assembly in Proxies for Atmospheric Aerosols. Nat. Commun. 2017, 8 (1), 1724 10.1038/s41467-017-01918-1. 29170428 PMC5701067

[ref2] Bowley E.; Liu W.; Adams D. J.; Squires A. M. Soft Materials with Time-Programmed Changes in Physical Properties through Lyotropic Phase Transitions Induced by PH-Changing Reactions. ACS Appl. Mater. Interfaces 2024, 16 (15), 19585–19593. 10.1021/acsami.4c01455. 38579106 PMC11040581

[ref3] Seddon J. M.; Templer R. H. Cubic Phases of Self-Assembled Amphiphilic Aggregates. Philos. Trans. R. Soc., A 1993, 344 (1672), 377–401. 10.1098/rsta.1993.0096.

[ref4] Qiu H.; Caffrey M. The Phase Diagram of the Monoolein/Water System: Metastability and Equilibrium Aspects. Biomaterials 2000, 21 (3), 223–234. 10.1016/S0142-9612(99)00126-X. 10646938

[ref5] Chung H.; Caffrey M. The Curvature Elastic-Energy Function of the Lipid–Water Cubic Mesophase. Nature 1994, 368 (6468), 224–226. 10.1038/368224a0. 8145820

[ref6] Lis L. J.; McAlister M.; Fuller N.; Rand R. P.; Parsegian V. A. Interactions between Neutral Phospholipid Bilayer Membranes. Biophys. J. 1982, 37 (3), 657–665. 10.1016/S0006-3495(21)00385-4. 7074191 PMC1328851

[ref7] Milsom A.; Squires A. M.; Laurence B.; Wo̅den B.; Smith A. J.; Ward A. D.; Pfrang C. Experimental Observation of the Impact of Nanostructure on Hygroscopicity and Reactivity of Fatty Acid Atmospheric Aerosol Proxies. Atmos. Chem. Phys. 2024, 24 (23), 13571–13586. 10.5194/acp-24-13571-2024.

[ref8] Richardson S. J.; Staniec P. A.; Newby G. E.; Rawle J. L.; Slaughter A. R.; Terrill N. J.; Elliott J. M.; Squires A. M. Glycerol Prevents Dehydration in Lipid Cubic Phases. Chem. Commun. 2015, 51 (57), 11386–11389. 10.1039/C5CC03771A. 26084976

[ref9] Sauerbrey G. The Use of Quartz Oscillators for Weighing Thin Layers and for Microweighing. Z. Fur. Phys. 1959, 155, 206–222. 10.1007/BF01337937.

[ref10] Höök F.; Kasemo B. The QCM-D Technique for Probing Biomacromolecular Recognition Reactions. In Piezoelectric Sensors; Springer Berlin Heidelberg: Berlin, Heidelberg, 2007, pp 425–447. 10.1007/978-3-540-36568-6_12.

[ref11] Niri V. H.; Flatt B. K.; Fakhraai Z.; Forrest J. A. Simultaneous Monitoring of Electroformation of Phospholipid Vesicles by Quartz Crystal Microbalance and Optical Microscopy. Chem. Phys. Lipids 2010, 163 (1), 36–41. 10.1016/j.chemphyslip.2009.10.004. 19883636

[ref12] Varineau P. T.; Buttry D. A. Applications of the Quartz Crystal Microbalance to Electrochemistry. Measurement of Ion and Solvent Populations in Thin Films of Poly(Vinylferrocene) as Functions of Redox State. J. Phys. Chem. 1987, 91 (6), 1292–1295. 10.1021/j100290a003.

[ref13] Ji Y.; Yin Z.-W.; Yang Z.; Deng Y.-P.; Chen H.; Lin C.; Yang L.; Yang K.; Zhang M.; Xiao Q.; Li J.-T.; Chen Z.; Sun S.-G.; Pan F. From Bulk to Interface: Electrochemical Phenomena and Mechanism Studies in Batteries via Electrochemical Quartz Crystal Microbalance. Chem. Soc. Rev. 2021, 50 (19), 10743–10763. 10.1039/D1CS00629K. 34605826

[ref14] Björklund S.; Kocherbitov V. Hydration-Induced Phase Transitions in Surfactant and Lipid Films. Langmuir 2016, 32 (21), 5223–5232. 10.1021/acs.langmuir.6b00452. 27124238

[ref15] Björklund S.; Kocherbitov V. Water Vapor Sorption-Desorption Hysteresis in Glassy Surface Films of Mucins Investigated by Humidity Scanning QCM-D. J. Colloid Interface Sci. 2019, 545, 289–300. 10.1016/j.jcis.2019.03.037. 30897425

[ref16] Björklund S.; Kocherbitov V. Humidity Scanning Quartz Crystal Microbalance with Dissipation Monitoring Setup for Determination of Sorption-Desorption Isotherms and Rheological Changes. Rev. Sci. Instrum. 2015, 86 (5), 055105 10.1063/1.4920919. 26026556

[ref17] Graf G.; Kocherbitov V. Determination of Sorption Isotherm and Rheological Properties of Lysozyme Using a High-Resolution Humidity Scanning QCM-D Technique. J. Phys. Chem. B 2013, 117 (34), 10017–10026. 10.1021/jp404138f. 23947953

[ref18] Johannsmann D. Viscoelastic Analysis of Organic Thin Films on Quartz Resonators. Macromol. Chem. Phys. 1999, 200 (3), 501–516. 10.1002/(SICI)1521-3935(19990301)200:3<501::AID-MACP501>3.0.CO;2-W.

[ref19] Milsom A.; Squires A. M.; Macklin J.; Wady P.; Pfrang C. Acoustic Levitation Combined with Laboratory-Based Small-Angle X-Ray Scattering (SAXS) to Probe Changes in Crystallinity and Molecular Organisation. RSC Adv. 2024, 14 (25), 17519–17525. 10.1039/D4RA01418A. 38818358 PMC11138859

[ref20] de Meyer F. J.-M.; Benjamini A.; Rodgers J. M.; Misteli Y.; Smit B. Molecular Simulation of the DMPC-Cholesterol Phase Diagram. J. Phys. Chem. B 2010, 114 (32), 10451–10461. 10.1021/jp103903s. 20662483

[ref21] Drabik D.; Chodaczek G.; Kraszewski S.; Langner M. Mechanical Properties Determination of DMPC, DPPC, DSPC, and HSPC Solid-Ordered Bilayers. Langmuir 2020, 36 (14), 3826–3835. 10.1021/acs.langmuir.0c00475. 32176506 PMC7467745

[ref22] Costigan S. C.; Booth P. J.; Templer R. H. Estimations of Lipid Bilayer Geometry in Fluid Lamellar Phases. Biochimica et Biophysica Acta (BBA) - Biomembranes 2000, 1468 (1–2), 41–54. 10.1016/S0005-2736(00)00220-0. 11018650

[ref23] Rand R. P.; Parsegian V. A. Hydration Forces between Phospholipid Bilayers. Biochimica et Biophysica Acta (BBA) - Reviews on Biomembranes 1989, 988 (3), 351–376. 10.1016/0304-4157(89)90010-5.

[ref24] Cowley A. C.; Fuller N. L.; Rand R. P.; Parsegian V. A. Measurement of Repulsive Forces between Charged Phospholipid Bilayers. Biochemistry 1978, 17 (15), 3163–3168. 10.1021/bi00608a034. 698192

[ref25] Parsegian V. A. Evidence for Long-Range Repulsion between Bimolecular Leaflets of Lecithin: An Examination of Structural Data. J. Theor. Biol. 1967, 15 (1), 70–74. 10.1016/0022-5193(67)90044-6. 6034162

[ref26] Richardson S. J.; Staniec P. A.; Newby G. E.; Terrill N. J.; Elliott J. M.; Squires A. M.; Góźdź W. T. Predicting the Orientation of Lipid Cubic Phase Films. Langmuir 2014, 30 (45), 13510–13515. 10.1021/la503313n. 25346159

[ref27] Nagle J. F.; Tristram-Nagle S. Structure of Lipid Bilayers. Biochimica et Biophysica Acta (BBA) - Reviews on Biomembranes 2000, 1469 (3), 159–195. 10.1016/S0304-4157(00)00016-2. 11063882 PMC2747654

[ref28] Kulkarni C. V.; Wachter W.; Iglesias-Salto G.; Engelskirchen S.; Ahualli S. Monoolein: A Magic Lipid?. Phys. Chem. Chem. Phys. 2011, 13 (8), 3004–3021. 10.1039/C0CP01539C. 21183976

[ref29] Barauskas J.; Landh T. Phase Behavior of the Phytantriol/Water System. Langmuir 2003, 19 (23), 9562–9565. 10.1021/la0350812.

[ref30] Chen Z.; Greaves T. L.; Fong C.; Caruso R. A.; Drummond C. J. Lyotropic Liquid Crystalline Phase Behaviour in Amphiphile–Protic Ionic Liquid Systems. Phys. Chem. Chem. Phys. 2012, 14 (11), 3825 10.1039/c2cp23698b. 22327439

[ref31] Demé B.; Zemb T. Hydration Forces between Bilayers in the Presence of Dissolved or Surface-Linked Sugars. Curr. Opin. Colloid Interface Sci. 2011, 16 (6), 584–591. 10.1016/j.cocis.2011.05.001.

[ref32] Schlaich A.; Daldrop J. O.; Kowalik B.; Kanduč M.; Schneck E.; Netz R. R. Water Structuring Induces Nonuniversal Hydration Repulsion between Polar Surfaces: Quantitative Comparison between Molecular Simulations, Theory, and Experiments. Langmuir 2024, 40 (15), 7896–7906. 10.1021/acs.langmuir.3c03656. 38578930 PMC11025125

[ref33] Rand R. P.; Fuller N. L.; Gruner S. M.; Parsegian V. A. Membrane Curvature, Lipid Segregation, and Structural Transitions for Phospholipids under Dual-Solvent Stress. Biochemistry 1990, 29 (1), 76–87. 10.1021/bi00453a010. 2322550

[ref34] Chung H.; Caffrey M. The Neutral Area Surface of the Cubic Mesophase: Location and Properties. Biophys. J. 1994, 66 (2), 377–381. 10.1016/S0006-3495(94)80787-8. 8161691 PMC1275705

[ref35] Templer R. H. On the Area Neutral Surface of Inverse Bicontinuous Cubic Phases of Lyotropic Liquid Crystals. Langmuir 1995, 11 (1), 334–340. 10.1021/la00001a056.

[ref36] Bain A.; Ghosh K.; Prisle N. L.; Bzdek B. R. Surface-Area-to-Volume Ratio Determines Surface Tensions in Microscopic, Surfactant-Containing Droplets. ACS Cent Sci. 2023, 9 (11), 2076–2083. 10.1021/acscentsci.3c00998. 38033804 PMC10683496

[ref37] Bzdek B. R.; Reid J. P.; Malila J.; Prisle N. L. The Surface Tension of Surfactant-Containing, Finite Volume Droplets. Proc. Natl. Acad. Sci. U. S. A. 2020, 117 (15), 8335–8343. 10.1073/pnas.1915660117. 32238561 PMC7165431

[ref38] Gérard V.; Noziere B.; Fine L.; Ferronato C.; Singh D. K.; Frossard A. A.; Cohen R. C.; Asmi E.; Lihavainen H.; Kivekäs N.; Aurela M.; Brus D.; Frka S.; Cvitešić Kušan A. Concentrations and Adsorption Isotherms for Amphiphilic Surfactants in PM 1 Aerosols from Different Regions of Europe. Environ. Sci. Technol. 2019, 53 (21), 12379–12388. 10.1021/acs.est.9b03386. 31553874

[ref39] Milsom A.; Squires A. M.; Ward A. D.; Pfrang C. The Impact of Molecular Self-Organisation on the Atmospheric Fate of a Cooking Aerosol Proxy. Atmos Chem. Phys. 2022, 22 (7), 4895–4907. 10.5194/acp-22-4895-2022.

[ref40] Latif M. T.; Brimblecombe P. Surfactants in Atmospheric Aerosols. Environ. Sci. Technol. 2004, 38 (24), 6501–6506. 10.1021/es049109n. 15669305

[ref41] Razak I. S.; Latif M. T.; Jaafar S. A.; Khan M. F.; Mushrifah I. Surfactants in Atmospheric Aerosols and Rainwater around Lake Ecosystem. Environmental Science and Pollution Research 2015, 22 (8), 6024–6033. 10.1007/s11356-014-3781-z. 25382497

[ref42] Nozière B.; Baduel C.; Jaffrezo J.-L. The Dynamic Surface Tension of Atmospheric Aerosol Surfactants Reveals New Aspects of Cloud Activation. Nat. Commun. 2014, 5 (1), 3335 10.1038/ncomms4335. 24566451 PMC3948073

