# Supplementary material for: Use of Humidity Controlled Quartz Crystal Microbalance with Simultaneous Grazing Incidence Small Angle X-ray Scattering to Investigate the Self-assembly and Energetics of Lipid Thin Films
Source: Langmuir. 2025 Apr 16;41(16):10216–22. doi: 10.1021/acs.langmuir.4c05158 (PMC12044696; doi:10.1021/acs.langmuir.4c05158)
Supplement: Supplementary file 1 — la4c05158_si_001.pdf [file la4c05158_si_001.pdf]

Supporting Information

The use of humidity controlled Quartz Crystal Microbalance with simultaneous Grazing Incidence Small Angle X-ray Scattering to investigate the self-assembly and energetics of lipid thin films

Author list

Jack Macklin[1], Christian Pfrang[2], Paul Wady[3], Wanli Liu[1], Ruaridh Davidson[4], Adam Milsom[2], Adam Squires[1]*

Author Address

[1] Department of Chemistry, University of Bath, South Building, Soldier Down Ln, Claverton Down, Bath, BA2 7AY, UK

[2] School of Geography, Earth and Environmental Sciences, University of Birmingham, Edgbaston, Birmingham, B15 2TT, UK

[3] Diamond Light Source, Diamond House, Harwell Science and Innovation Campus, Didcot, OX11 0QX, UK

[4] School of Chemistry, University of Bristol, Cantock's Close, Bristol, BS8 1TS, UK

* Email: as3474@bath.ac.uk

Table of contents

Figure Number	Details
S1	Identification of the peaks caused by the diffraction of the specular reflected beam
S2	Incident angle-dependent 2D patterns
S3	GI-SAXS background from blank sample

Identification of the peaks caused by the diffraction of the specular reflected beam

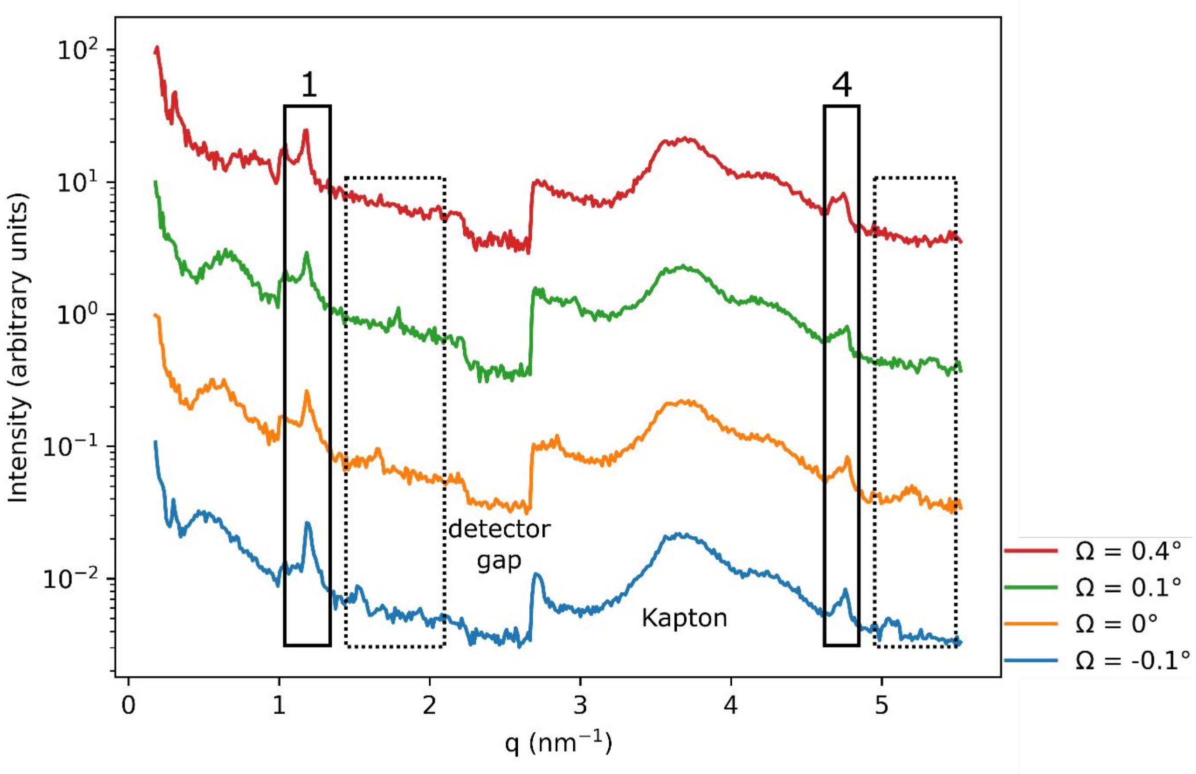

Figure S1: Integrated 1D GI-SAXS patterns collected from a thin film of DMPC spin-coated onto a QCM wafer. The angle of the GI-SAXS stage, Ω, was set in turn to Ω = -0.1˚ (blue line, bottom), Ω = 0˚ (orange line, second), Ω = 0.1˚ (green line, third) and Ω = 0.4˚ (red line, top). Ω = 0˚ corresponds to an incident angle of approximately zero (+/- 0.2˚). The first and fourth order lamellar reflection are numbered and indicated by a solid box. Peaks due to scattering from the specular reflected beam are indicated by a dotted box and are identified by their variation as the

angle on incidence of the beam is varied. All patterns were collected at 80% RH. The broad peak at 3.8 nm^{-1} is due to Kapton, and the low scattering around 2.3 nm^{-1} is from the gap in the detectors.

Incident angle-dependent 2D patterns

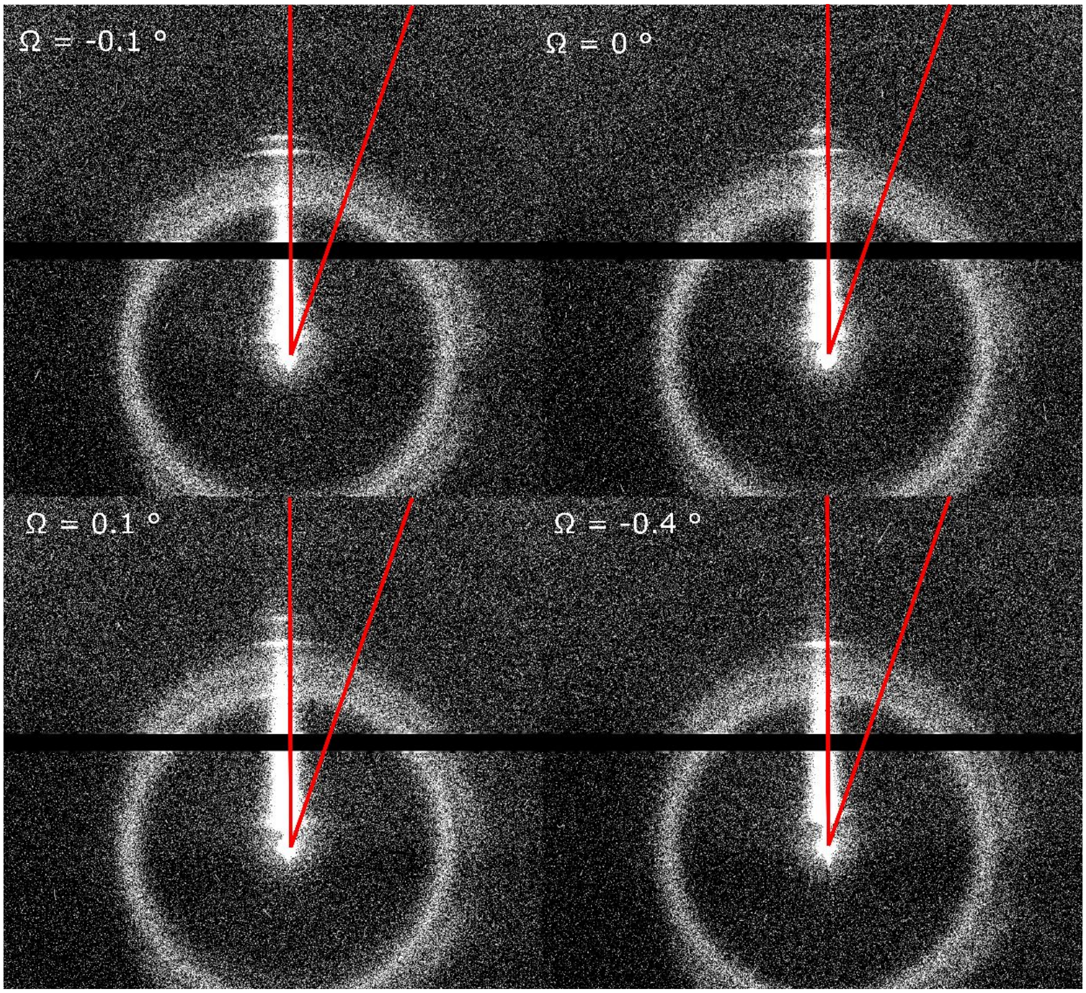

Figure S2: The 2D patterns from which the 1D patterns presented in Figure S1 were calculated.

The red wedge shows the region of interest over which the 1D pattern was integrated

GI-SAXS background from blank sample

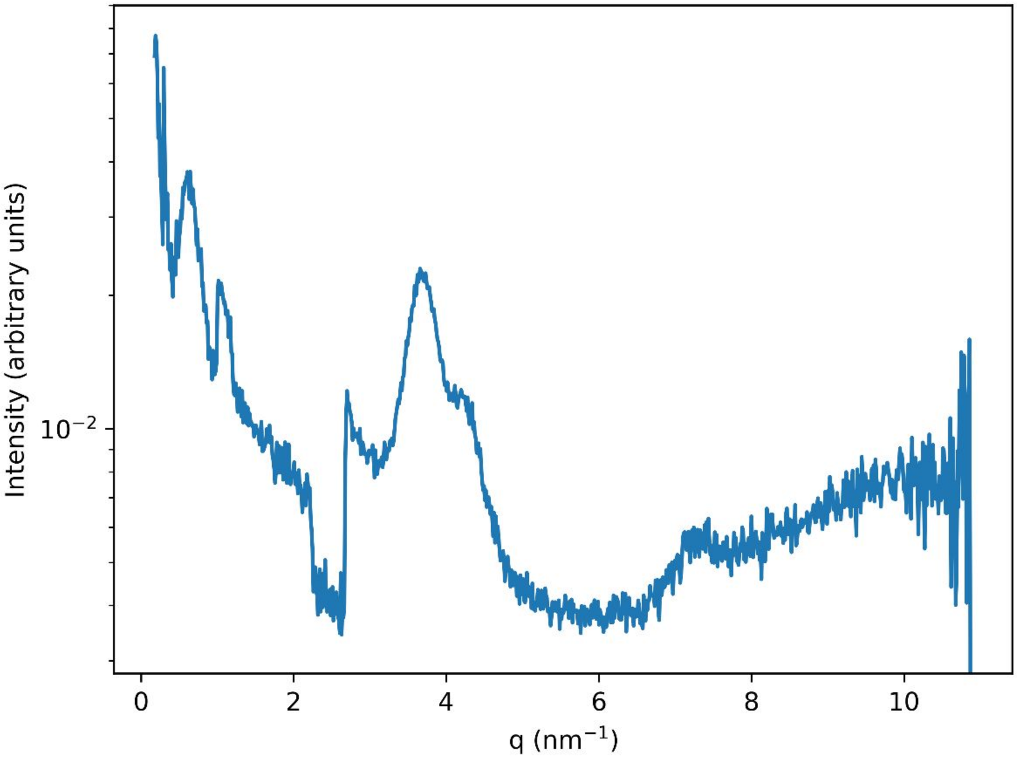

Figure S3: The background scattering from the GI-SAXS measurements. This is the pattern resulting from a blank QCM wafer with nothing coated on it, showing the artefacts that we see in the various GI-SAXS patterns such as the detector gap and the Kapton hump.
